# Spatial pattern of severe acute respiratory syndrome in-out flow in 2003 in Mainland China

**DOI:** 10.1186/s12879-014-0721-y

**Published:** 2014-12-31

**Authors:** Chengdong Xu, Jinfeng Wang, Li Wang, Chunxiang Cao

**Affiliations:** LREIS, Institute of Geographic Sciences and Natural Resources Research, Chinese Academy of Sciences, Beijing, 100101 China; Key Laboratory of Surveillance and Early Warning on Infectious Disease, Chinese Center for Disease Control and Prevention, Beijing, 102206 China; Institute of Remote Sensing and Digital Earth, Chinese Academy of Sciences, Beijing, 100094 China

**Keywords:** In-out flow, Mainland China, SARS

## Abstract

**Background:**

Severe acute respiratory syndrome (SARS) spread to 32 countries and regions within a few months in 2003. There were 5327 SARS cases from November 2002 to May 2003 in Mainland China, which involved 29 provinces, resulted in 349 deaths, and directly caused economic losses of $18.3 billion.

**Methods:**

This study used an in-out flow model and flow mapping to visualize and explore the spatial pattern of SARS transmission in different regions. In-out flow is measured by the in-out degree and clustering coefficient of SARS. Flow mapping is an exploratory method of spatial visualization for interaction data.

**Results:**

The findings were as follows. (1) SARS in-out flow had a clear hierarchy. It formed two main centers, Guangdong in South China and Beijing in North China, and two secondary centers, Shanxi and Inner Mongolia, both connected to Beijing. (2) “Spring Festival travel” strengthened external flow, but “SARS panic effect” played a more significant role and pushed the external flow to the peak. (3) External flow and its three typical kinds showed obvious spatial heterogeneity, such as self-spreading flow (spatial displacement of SARS cases only within the province or municipality of onset and medical locations); hospitalized flow (spatial displacement of SARS cases that had been seen by a hospital doctor); and migrant flow (spatial displacement of SARS cases among migrant workers). (4) Internal and external flow tended to occur in younger groups, and occupational differentiation was particularly evident. Low-income groups of male migrants aged 19–35 years were the main routes of external flow.

**Conclusions:**

During 2002–2003, SARS in-out flow played an important role in countrywide transmission of the disease in Mainland China. The flow had obvious spatial heterogeneity, which was influenced by migrants’ behavior characteristics. In addition, the Chinese holiday effect led to irregular spread of SARS, but the panic effect was more apparent in the middle and late stages of the epidemic. These findings constitute valuable input to prevent and control future serious infectious diseases like SARS.

**Electronic supplementary material:**

The online version of this article (doi:10.1186/s12879-014-0721-y) contains supplementary material, which is available to authorized users.

## Background

Humans have been exposed continually to newly emerged infectious diseases [1]-[3], especially 1918 influenza, 2003 severe acute respiratory syndrome (SARS), 2009 H1N1 influenza, 2012 novel coronavirus, 2013 H7N9 influenza pandemics and Ebola virus in 2014. Without exception, these viruses were harbored in an animal reservoir and jumped the species barrier to infect humans, presenting a serious threat to human health. SARS, “the Black Death in the 21st century”, spread to 32 countries and regions worldwide within a few months [4]. Globally, there were 8096 cases of SARS and 774 deaths, with 5327 cases in Mainland China, involved 29 provinces, resulted in 349 deaths [4], and caused total economic losses of $18.3 billion, which accounted for 1.3% of the gross domestic product in Mainland China [5].

There have been many studies on virus transmission during the SARS epidemic, which have included three main aspects. (1) Use of the Susceptible–Infected, Susceptible–Infected–Removed or Susceptible–Exposed–Infectious–Recovered model of infectious diseases [6]-[11]. For example, Li et al. introduced the SI model and piecewise SI model to forecast the cumulative number of cases of SARS in Beijing. The piecewise SI model showed the change point on April 21, 2003. Wang et al. used the SIR or SEIR model to study SARS transmission in Beijing, Hong Kong or Singapore. Their results showed that public health interventions such as early recognition, prompt isolation, and appropriate precautionary measures, could effectively limit spread of the virus. (2) Use of spatial statistics to explore the spatial clustering characteristics of SARS [12]-[14]. For example, some researchers used geostatistic, such as semivariogram, Moran’s I and LISA statistics to study the risks of SARS transmission and spatiotemporal evolution in Beijing or Guangzhou. This provided a scientific basis for the emergency plan for the outbreak of SARS or other unexpected new epidemics in urban areas. (3) Use of dots diffusion model to study spread of the SARS epidemic by different modes of transport, and use flying spot spread model to study input–output sources of the spread of SARS in different regions [15],[16], to predict trends in the spread of SARS in the medium to long term on a national and metropolitan basis. Many studies have explored the transmission of the SARS epidemic from the affected to neighboring areas [7]-[11],[17]-[21], such as neighborhoods, hospitals, and other cities. These studies were good at reflecting the spatial diffusion of SARS in the local area, but not at an interprovincial level.

Information on the SARS in-out flow transmission at provincial level in Mainland China helps us to understand the temporospatial spread of infectious diseases like SARS [22]. This provides a good reference for prevention of similar infectious disease outbreaks in the future. To reflect the interprovincial diffusion paths and characteristics of the SARS epidemic, the present study used in-out flow data at provincial and municipal level to study the spatial spread of the SARS epidemic in Mainland China. Instead of single spatial location studies in the current most population epidemiological literature, we used in-out flow model to explore the interregional transmission of the disease. This can better explain the spatiotemporal evolution in the process of disease transmission in the country.

## Methods

### SARS data

Formal ethical approval was not required for the study because all patient-identifiable fields were removed and the statistical analysis on the population was applied.

**Figure 1:**
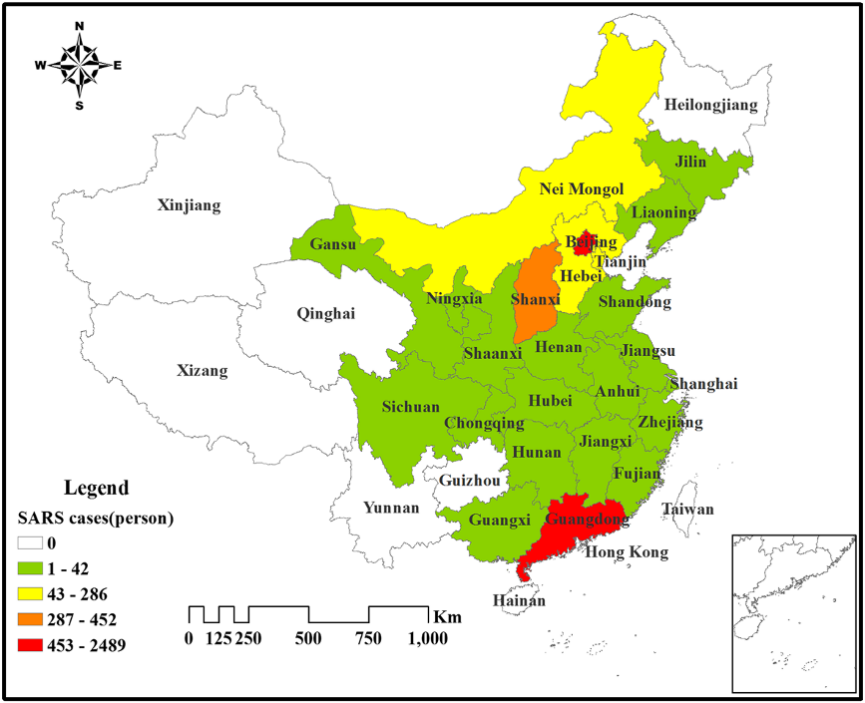
**Distribution of SARS cases during 2002–2003 in Mainland China.**

The SARS data from November 2002 to May 2003 in Mainland China were provided by the Chinese Center for Disease Control and Prevention. There were a total of 5327 cases of SARS (Figure 1), with each person a record in the dataset. The attribute items in the dataset included sex, age, occupation, registered residence, workplace or current residence, onset location, reporting units, onset time, hospitalization time, diagnosis time, and other information, which was separate information collected from the SARS case. Registered residence refers to the “hukou” address, is the address of a person’s ID card, and reflects the origin of the floating population. Permanent residence refers to living in a place for more than six months. Current residence is almost consistent with permanent residence, but easier to change than permanent residence. Workplace refers to the location of a person’s job. Onset location refers to the approximate place of SARS onset. Medical location refers to the location of treatment SARS, and the medical unit is an officially designated hospital.

SARS in-out flow occurred between permanent residence, onset location and medical location. According to the definition of in-out flow, transmission of SARS cases in Mainland China was divided into internal flow and external flow. External flow was further divided into self-spreading, hospitalized, and migrant flows. They could be interpreted by epidemiology (Additional file 1: Table S1). Permanent residence is based on current residence, but if there is no current residence, workplace is used instead; onset location is based on place of SARS onset; and medical location is based on the reporting location.

SARS data processing followed three steps. (1) Extraction of valid records. We only used three places (permanent residence, onset location and medical location) with complete spatial location information, that is, if one of these pieces of information was missing, the record was removed. (2) Determining the three types of spatial location information at the provincial or municipal level. (3) Selecting data with the three spatial locations within the same province/municipality as internal flow (also called provincial flow), or data with the different provinces/municipalities as external flow (also called interprovincial flow). The external flow was subdivided into self-spreading flow, hospitalized flow and migrant flow. Self-spreading flow represented a typical external flow, with spatial displacement of SARS cases only within the province or municipality of onset and medical locations. Hospitalized flow indicated a typical kind of external flow, with spatial displacement of SARS cases that had been seen by a hospital doctor. Migrant flow was a typical kind of external flow with spatial displacement of SARS cases among migrant workers, and got sick but had to be treated in their hometown. The flow was not only applicable to patients who were rural migrant workers, but also included a small number of people with other occupations. For example, 41.5% of migrant flow was related to migrant workers (people seek work to earn money in the non-harvest season, and the salary is their main income for the year), 1.2% of migrant flow was related to workers (individuals without production materials, rely on the pay by being employed in manual or technical work), and 3.7% of migrant flow was related to public staff. Take Beijing as permanent residence for example, if people are in the permanent population in Beijing, and their onset and medical location locations are also in Beijing, they belong to provincial flow. If people are non-permanent population in Beijing, but their onset and medical location locations are in Beijing, they belong to self-spreading flow. If people are non-permanent population in Beijing, and their onset location is also not in Beijing, but their medical location is in Beijing, they belong to hospitalized flow. If people are in the permanent population in Beijing, their onset location is not in Beijing, but their medical location is still in Beijing, they belong to migrant flow. Following the above three steps of data processing, and elimination of invalid records (Additional file 3: Table S3), we finally obtained 1776 cases of SARS internal flow, which accounted for 90% of SARS in-out flow data; and 198 cases of SARS external flow, accounting for 10% of in-out flow data, including 101 cases of self-spreading flow, 15 of hospitalized flow, and 82 cases of migrant flow. The 1974 cases having complete address records are a sample of the total 5327 SARS cases in the country. The data’s frequency distributions of SARS flow and total cases agree well with each other in various dimensions, such as gender, age and occupation (Additional files 5, 6, 7: Figures S8-10). Therefore, there is no evidence there is systematic bias of the sampling, and the missing data were updated by ratio estimator method (see Additional file 8).

### Interpretation of SARS in-out flow

Input–output flow came from the spatial location changes of infected individuals as different epidemic characteristics. For example, SARS patients moved from their permanent residence to the infection location after infected, and then moved to the onset location when their symptoms appeared. Finally, their location moved to the reporting unit where they received treatment. Therefore input flow refers to the permanent population infected in other place, but returned to the permanent location for treatment. Output flow refers to the non-permanent population infected in permanent place, but moved to other place for treatment. For example, if a person is the permanent population in Beijing, his/her onset location in Hebei province, or he/she chooses to be treated in Beijing, he/she is classified as input flow for Beijing. If a person is not the permanent population in Hebei, his/her onset location in Hebei, or he/she chooses to be treated in Beijing, he/she is classified as output flow for Hebei province.

SARS in-out flow is defined to explore the input–output transmission mechanism of the infected patient and the material and information flows in the spatial location transformation at the provincial scale.

### Construction of SARS in-out flow model

The SARS in-out flow model used the multiple spatial locations to explore the migration path of SARS at the provincial or municipal level. The degree and clustering are known as transitivity, and both are typical properties of acquaintance networks [23]. Where the degree *L*_*i*_ of a node *i* is obtained by counting the actual number of edges between different nodes [24], clustering coefficient *C*_*i*_ describes information and material flow [22]. We constructed a SARS in-out flow network model by the actual connection edges *L*_*i*_ between provinces and local clustering coefficient *C*_*i*_, to measure the clustering phenomenon of SARS flow transmission in Mainland China. The formula is:

1 $$L_{i}=\sum_{j\in N}l_{ij}$$

where *L*_*i*_ was defined as the total degree ($L_{i}=L_{i}^{\mathrm{in}}+L_{i}^{\mathrm{out}}$), the in-degree of the node; $L_{i}^{\mathrm{in}}$ referred to the number of ingoing links between different regions, and the out-degree of the node $L_{i}^{\mathrm{out}}$ referred to the number of outgoing links. *i* and *j* referred to the different nodes in provinces or municipalities, and *N* represented the number of provinces or municipalities in Mainland China (1 ≤ *N* ≤ 34).*T*he greater *L*_*i*_, the easier SARS cases spread.

Local clustering coefficient *C*_*i*_ is defined as:

2 $$C_{i}=\frac{\sum_{i=n,j=n}Case_{ij}}{\sum_{i=N,j=N}Case_{ij}}$$

where *n* was the edges of the directed network, taking one node as the center; *N* was the total number of edges of the directed network; *Case*_*ij*_ referred to SARS cases carrying the material flow with the corresponding information flow by directed network transmission from the center *i* to *j*; and *C*_*i*_ described the aggregation level of the material flow and information flow between the different provinces or municipalities.

In particular, super-spread events (SSEs) will lead to localized outbreaks, and it is essential to understand and quantify these SSEs against other infectious diseases similar to SARS [25]. The relationship among these cross-regional infected people is mainly studied by SSEs of external flow.

### Flow mapping

Flow mapping, as a spatial visualization exploratory method, is typically used to visualize spatial interaction data, such as population migration and disease transmission [26],[27]. In the flow map, origins and destinations are connected to each other by a straight or curved line. Arrows represent the direction from the source to the destination, and line width and color symbolizes the flow. In this study, flow mapping was based on the regional structure of SARS in-out flow from the source to the destination, and mainly displayed the material and information flows carried by the SARS cases.

## Results

### Hierarchy of SARS in-out flow

**Figure 2:**
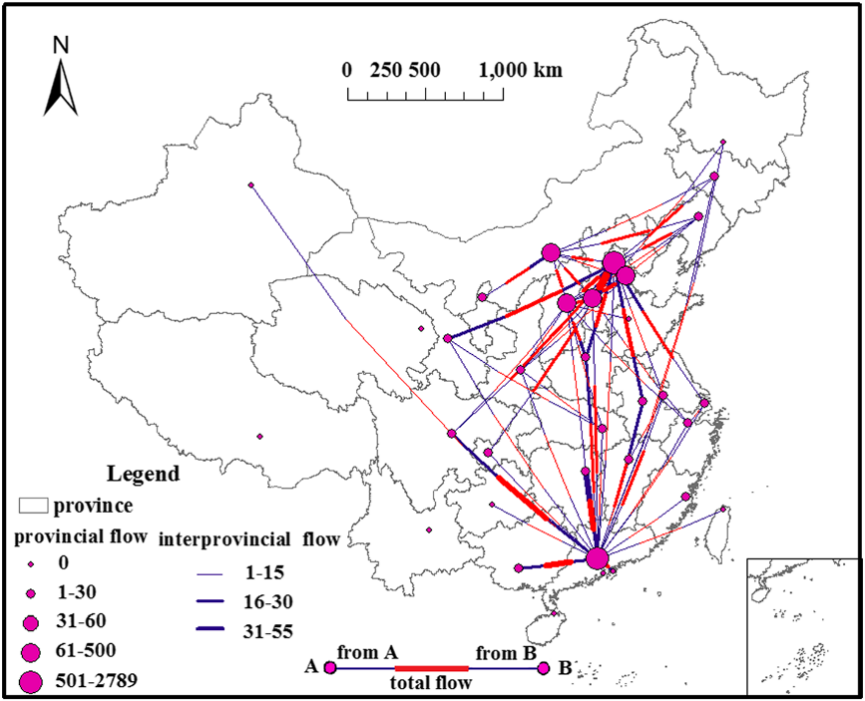
**SARS in–out flow among the provinces of China.**

**Figure 3:**
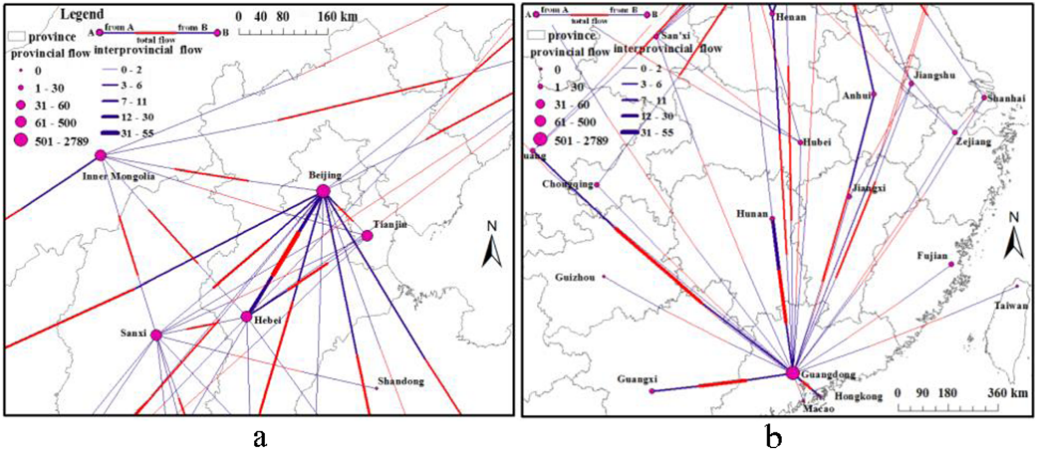
**SARS in–out flow (a) between Beijing and its neighboring provinces, and (b) between Guangdong and its neighboring provinces.**

Generally, the floating population in China always moves from the underdeveloped regions to the relatively developed provinces. However, there was a clear hierarchy of SARS in-out flow, which formed two major centers determined by the local clustering coefficient. The “hierarchy” representing SARS in-out flow cases formed the different levels of the cluster center, and Guangdong and Beijing were at the highest level of cluster center (Figure 2) during SARS transmission and formed the first hierarchy. Shanxi and Inner Mongolia were at the secondary level of the cluster centers in underdeveloped provinces (Figure 3), and formed the second hierarchy.

Specifically, the overall SARS in-out flow showed significant spatial clustering, with the first cluster in Guangdong, and the second in Beijing and surrounding areas (such as Shanxi, Inner Mongolia, Hebei, and Tianjin). Based on whether the intervention policy of the Chinese government was implemented, the whole SARS outbreak period was divided into two major segments (Additional file 2: Table S2). Cluster locations without intervention policy were in Guangdong, Shanxi and Inner Mongolia, while those with intervention were in Shanxi, Hebei, Tianjin, Inner Mongolia, and Beijing.

Besides migrants in China moved to the areas with an expected high income, more factors affect SARS transmission, such as the Chinese New Year and seasonal changes such as the crop harvest period resulted in a return migratory flow, but this was relatively dispersed throughout the country, such as Sichuan, Anhui, Hunan, Hebei, Shanxi and Inner Mongolia.

### Significance of SARS panic effect

“Spring Festival travel” increased the external flow between Guangdong and its neighboring provinces, but the “SARS panic effect” played a more significant role in SARS in-out flow, and pushed the external flow towards its peak. Specifically, “Spring Festival travel” which peaked in February 2003, caused SARS to spread randomly in the early period, and increased the external flow by 7.1% compared with before February 2003. The “SARS panic effect” peaked in April 2003, and in the mid-period the external flow increased by 49.5% compared with before April 2003.

**Figure 4:**
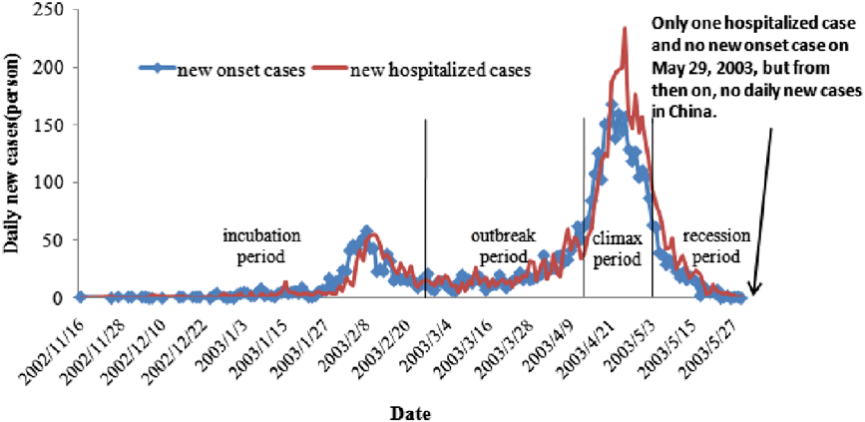
**Daily new SARS cases in Mainland China.**

Throughout the SARS outbreak (Figure 4), the floating population in Mainland China was subject to the Spring Festival effect, but the panic effect was particularly significant for migration. Specifically, the onset of the external flow in Mainland China initially focused on the weeks before and after the Spring Festival, which stimulated massive population flows, commonly known as “Spring Festival transportation”. Owing to an initial misunderstanding of the strong spread and serious harm of the SARS virus, panic induced massive, unprecedented population flows within a short time in China in mid-2003.

Hospital admission for SARS during the Spring Festival did not attract much attention. However, the spread of the SARS virus due to panic migration in mid-April turned to be serious on April 20, 2003. The time lag between onset and admission provided the temporal and spatial feasibility of SARS virus transmission in various forms, such as within families, communities, hospitals and clinics, stochastic diffusion within one city, and long-distance transmission.

### Heterogeneity of external flow

There was obvious spatial heterogeneity in the external flow.

The first flow referred to SARS cases that resulted from the external flow from the permanent residence to onset location, which mainly occurred within a small range, within families and neighborhoods as the key spatial location of the SARS outbreak and person-to-person transmission.

**Figure 5:**
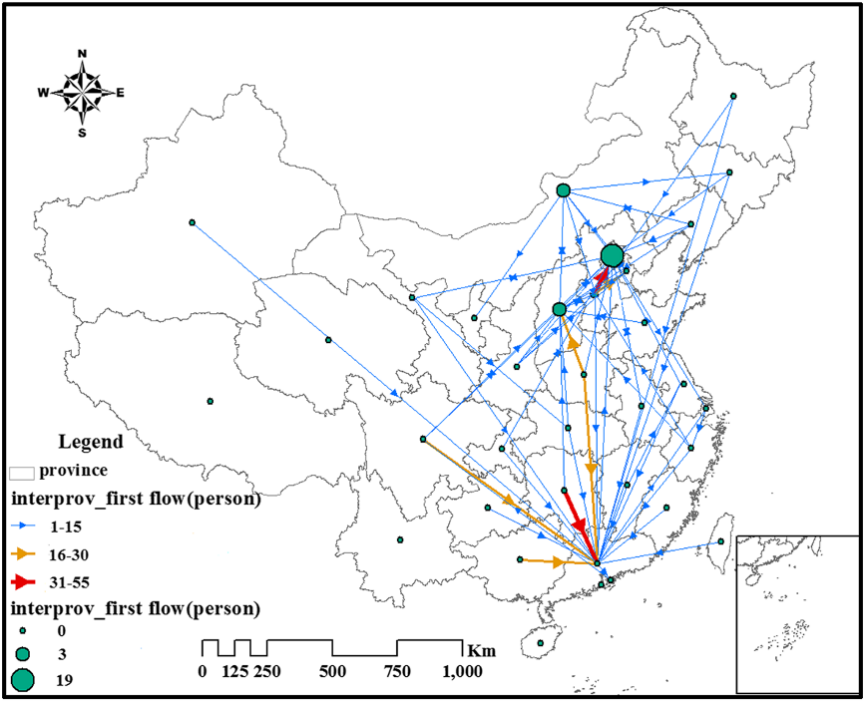
**The first external flow of SARS.**

The first external flow of SARS (Figure 5) was mainly from Hebei to Beijing, and Hunan to Guangdong, accounting for 12.6% and 8.6% of cases, respectively. This was followed by the first external flow from Guangxi to Guangdong, Sichuan to Guangdong, Hebei to Tianjin, within Beijing, Henan to Shanxi, and Henan to Guangdong, which accounted for a total of 19.7% of cases. In addition, the external flow from Guangdong to Hong Kong, Taiwan to Guangdong, and Hong Kong to Sichuan, accounted for 2.0%, 0.5% and 0.5% of cases, respectively. The population distribution of the first external flow of SARS in the remaining provinces was more dispersed, especially in Tibet, Qinghai, Yunnan and Hainan; there was no first external flow of SARS.

The second external flow referred to SARS cases that resulted from the external flow from the onset location to medical location, which mainly occurred within a small range, with hospitals as the key spatial location of the SARS outbreak and person-to-person transmission.

**Figure 6:**
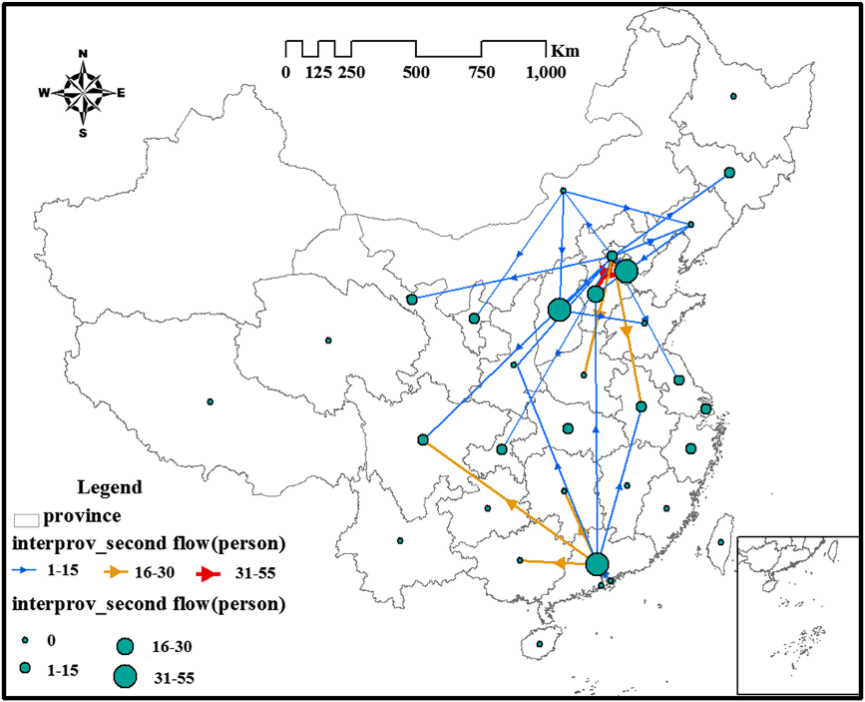
**The second external flow of SARS.**

The second external flow of SARS (Figure 6) was mainly within Guangdong, from Beijing to Hebei, and within Shanxi, accounting for 18.7%, 15.2% and 10.6% of cases, respectively. This was followed by the second external flow within Tianjin and Hebei, and from Guangdong to Guangxi, and Guangdong to Sichuan, accounting for 5.6%, 5.1%, 4.5% and 3.5% of cases, respectively. In addition, the second external flow of SARS from Hong Kong to Guangdong accounted for 2.0% of cases. The population distribution of the second external flow of SARS in the remaining provinces was more sporadic, especially in Xinjiang, Tibet, Qinghai, Yunnan, Guizhou, Hubei, Hainan, Jiangxi, Fujian, Taiwan, Zhejiang and Heilongjiang, there was no second external flow of SARS.

From the route of SARS transmission and the material flow, the first population flow was mainly from Hebei to Beijing, and Hunan to Guangdong, and the second flow was mainly within Guangdong, from Beijing to Hebei, and within Shanxi. Beijing and Hong Kong were the two transmission hubs for SSEs, with Beijing playing a central role in the spread of SARS in inland provinces, and Hong Kong playing a central role in the global transmission of SARS (Additional file 4: Figure S1).

The three typical types of SARS external flow are shown in Additional file 4: Figures S2-S7. The first self-spreading flow was mainly from Hunan to Guangdong, Hebei to Tianjin, and Henan to Shanxi. The second self-spreading flow was mainly within Guangdong, Shanxi, Tianjin and Hebei. The first hospitalized flow was mainly within Beijing. The second hospitalized flow was mainly from Beijing to Hebei, and Anhui to Shanxi. The first migrant flow was mainly from Hebei to Beijing, Guangxi to Guangdong, Sichuan to Guangdong, and Hunan to Guangdong. The second migrant flow was mainly from Beijing to Hebe, Guangdong to Guangxi, and Sichuan to Hunan.

### Strata of external flow

Internal and external flows tended to occur more frequently in younger age groups, and occupational differentiation was particularly evident. Specifically, low-income groups of male migrants aged 19–35 years were the main avenues of external flow.

During the SARS outbreak, there were slightly more women than men in the gender distribution of provincial flow, but there were more men in the interprovincial flow. Occupational differentiation was significant, with medical personnel being the highest for internal flow, while migrant workers and students were the highest for external flow.

Before implementation of the intervention policy, medical staff was still the most prominent occupation in provincial flow, but it was significantly reduced after implementation of the intervention policy. Migrant workers, students and peasants were the highest occupation groups in external flow. However, owing to the enhanced prevention awareness, medical staff disappeared from the occupational distribution of external flow.

**Figure 7:**
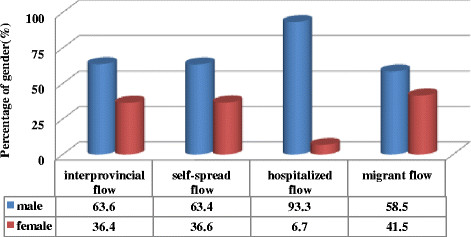
**Gender distribution of SARS external flow.**

Figure 7 shows that men accounted for 63.6% of SARS external flow, which was 27.2% higher than women. For self-spreading flow, men were responsible for 26.7% more SARS transmission than women. For hospitalized flow, men were responsible for 86.7% more transmission than women. For migrant flow, men accounted for 17.0% more transmission than women.

**Figure 8:**
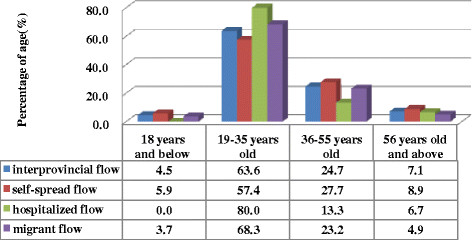
**Age distribution of SARS external flow.**

From the age distribution (Figure 8), most of the SARS external flow was in young adults aged 19–35 years. This age group accounted for 63.6% of the external flow, compared with only 24.7% in the 36–55 years age group. For self-spreading flow, the 19–35 years age group accounted for 57.4%, compared with 27.7% in the 36–55 years age group. For hospitalized flow, the 19–35 years age group accounted for 80.0%, compared with 13.3% in the 36–55 years age group. For migrant flow, the 19–35 years group accounted for 68.3%, compared with 23.2% in the 36–55 years age group.

**Figure 9:**
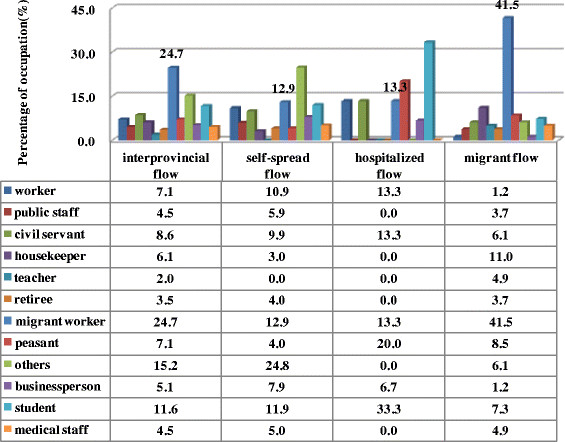
**Occupational distribution of SARS external flow.**

From the occupational distribution (Figure 9), low-income groups were the main route of SARS external flow. Low-income groups referred to the grassroots and students, mostly from rural areas. Overall, low-income groups accounted for 61.1%, grassroots workers for 49.5%, migrants for 24.7%, and students for 11.6%. For self-spreading flow, low-income groups accounted for 37.6%, grassroots workers for 25.7%, migrant workers for 12.9%, and students for 11.9%. For hospitalized flow, low-income groups accounted for 66.7%, grassroots workers for 33.3%, and students for 33.3%. For migrant flow, low-income groups accounted for 72.0%, grassroots workers for 64.6%, migrant workers for 41.5%, and students for 7.3%.

## Discussion

This study applied a SARS in-out flow model and flow mapping to visualize and explore the spatial migration path of SARS at the provincial or municipal level. We demonstrated that SARS in-out flow played an important role in nationwide transmission of the disease. There are three findings in the study. (1) SARS in-out flow moved from the underdeveloped populous provinces to the relatively developed regions, the proximity transmission and “Fly Dots” spreading along transportation coexisted during the epidemic period. (2) Irregular flow of infectious diseases like SARS was influenced by people’s behavioral characteristics and Chinese traditional festivals, but more apparent by the panic effect in the middle and late stages of the epidemic. (3) Interprovincial flows were dominated by young male migrant workers. They were both the main rural surplus labor and potential urban residents in the medium and small cities of China.

Hu et.al. [22] have explored the epidemic transmission network of SARS in-out flow in mainland China, which aimed to find the spatiotemporal evolution pattern of the individual location and transformation during the SARS epidemic. The present study focused on different objectives, which included the spatial pattern of transformation in the SARS epidemic based on the three individual locations (permanent residence, onset location and medical location), spatial transmission characteristics of three typical kinds of external flow, and the spatial transmission characteristics of SSEs. Hu et al. indicated that Guangdong and Beijing were two centers for the transmission of SARS in the early and mid-late period. In addition, the output network had higher-intensity spread capacity and larger influence range than the input network. Although the present study had broader and deeper findings, for example, the spatial pattern of transmission, the results showed that the direction of flow was mainly from the underdeveloped to the developed regions. There was also “Fly Dots” spreading by transportation, and Chinese traditional festivals combined with a panic effect strengthened the transmission, and the transmission was mainly by the young male migrant workers.

There were some limitations to our study. (1) The SARS in-out flow model needed information about permanent residence, onset location and medical location, so that the pathways could be studied for the spatial location transformation of infected individuals, which was a relatively high requirement for data. (2) There were actually 983 cases of SARS among medical staff, which accounted for 18.5% of the total number of cases. The SARS cases among medical staff always occurred within a small range of locations, such as hospitals and clinics in the same provinces or cities, that belonged to the provincial flow. SARS in-out flow was only focused on interprovincial flow, and not provincial flow. Therefore, the effect of nosocomial transmission was neglected. (3) The division of the three groups was a new mixed proposed for this study, which depended on the distinguishing features of key spatial location changes of individuals as well as their attendance at common social functions. In addition, the location information in the dataset was used to the greatest extent. The dataset had no detailed epidemiological information, for example, the movement of SARS patients, which gave some uncertainty to the results. The SARS in-out flow model only reflected the real interprovincial flow of SARS cases. In fact SARS in-out flow is influenced by many socioeconomic factors. In-depth research should take account of more complex models and variables, such as using the gravity model to study SARS flow data, obtain the relevant parameters, and conduct analysis and prediction [28],[29]; and using small-world networks to study the high agglomeration phenomena appearing in SARS in-out flow data.

The results of our study will help to prevent and control infectious diseases similar to SARS in the future. For example, one countrywide health insurance card should be actively explored. The new policy is for low-income groups of migrants to be protected against infectious diseases, which helps to prevent them spreading from the central cities to the marginal rural areas. This is important for China, because it is in a period of urbanization, with large-scale migration of the population that could last for several decades.

## Conclusions

In conclusion, SARS in-out flow played an important role in nationwide transmission of the disease during 2002-2003. The flow had obvious spatial heterogeneity, which was influenced by migrants’ behavior characteristics. In addition, the Chinese holiday effect led to irregular spread of SARS, but the panic effect was more apparent in the middle and late stages of the epidemic. These findings constitute valuable input to prevent and control future serious infectious diseases like SARS.

## Additional files

## Electronic supplementary material

Additional file 1: Table S1.: Epidemiological interpretation of SARS in-out flow data. (DOC 46 KB)

Additional file 2: Table S2.: Effect of SARS intervention policy in China. (DOC 44 KB)

Additional file 3: Table S3.: Sample size of processed data at the provincial level. (DOC 66 KB)

Additional file 4: **Interpretation of typical types of SARS external flow.** (**Figure S1.** SARS SSEs of SARS external flow. **Figure S2.** The first self-spreading flow of SARS. **Figure S3.** The second self-spreading flow of SARS. **Figure S4.** The first hospitalized flow of SARS. **Figure S5.** The second hospitalized flow of SARS. **Figure S6.** The first migrant flow of SARS. **Figure S7.** The second migrant flow of SARS.). (DOC 701 KB)

Additional file 5: Figure S8.: Frequency distributions of the SARS flow and total SARS cases by gender. (DOC 50 KB)

Additional file 6: Figure S9.: Frequency distributions of the SARS flow and total SARS cases by age. (DOC 52 KB)

Additional file 7: Figure S10.: Frequency distributions of the SARS flow and total SARS cases by occupations. (DOC 59 KB)

Additional file 8: Ratio estimator is sampling with proportional to aggregate size, which is used to update the total in-out flow. (DOC 34 KB)

Below are the links to the authors’ original submitted files for images.

Authors’ original file for figure 1

Authors’ original file for figure 2

Authors’ original file for figure 3

Authors’ original file for figure 4

Authors’ original file for figure 5

Authors’ original file for figure 6

Authors’ original file for figure 7

Authors’ original file for figure 8

Authors’ original file for figure 9
