# Supplementary material for: Spatial pattern of severe acute respiratory syndrome in-out flow in 2003 in Mainland China
Source: BMC Infect Dis. 2014 Dec 31;14:721. doi: 10.1186/s12879-014-0721-y (PMC4322810; doi:10.1186/s12879-014-0721-y)
Supplement: Supplementary file 1 — Additional file 1: Table S1.: Epidemiological interpretation of SARS in-out flow data. (DOC 46 KB) [file 12879_2014_721_MOESM1_ESM.doc]

**Table S1.** Epidemiological interpretation of SARS in–out flow data

| **Type** | | **Type indicators** | | | **Corresponding epidemiological interpretation** |
| --- | --- | --- | --- | --- | --- |
|  |  | Person in permanent residence | Onset location = permanent residence | Medical location = permanent residence |  |
| Internal flow | Provincial flow | yes | yes | yes | SARS case with all three pieces of spatial location information, only spread within the province or municipality, but without virus output. |
| External flow (interprovincial flow) | Self-spreading flow | no | yes | yes | SARS cases are non-permanent population, same onset and medical locations, but both different from permanent residence, only spread between onset and medical location, also without virus output. |
|  | Hospitalized flow | no | no | yes | SARS cases are non- permanent population, but with onset and medical location different, viruses spread taking onset and medical location, with virus in-output, prevention objects should be focused on medical location. |
|  | Migrant flow | yes | no | yes | SARS cases are permanent population, with same permanent residence and medical location, but both different from onset location. Viruses spread among the three spatial locations, with virus in/output, defense objects are focused on permanent residence. |
|  | Other types | – | – | – | Other types of external flow have no related statistics. |

To simplify different types of flow indicators, “yes” represents spatial location the same as permanent location, “no” represents spatial location different from permanent location, “–” means missing data of this type.
