# Supplementary material for: Spatial pattern of severe acute respiratory syndrome in-out flow in 2003 in Mainland China
Source: BMC Infect Dis. 2014 Dec 31;14:721. doi: 10.1186/s12879-014-0721-y (PMC4322810; doi:10.1186/s12879-014-0721-y)
Supplement: Supplementary file 2 — Additional file 2: Table S2.: Effect of SARS intervention policy in China. (DOC 44 KB) [file 12879_2014_721_MOESM2_ESM.doc]

**Table S2 Effect of SARS intervention policy in China**

| **Flow type** | **Cluster center** | **Cluster location** **without intervention** | **Cluster location**  **with intervention** | **Effect of**  **intervention policy** |
| --- | --- | --- | --- | --- |
| SARS in–out flow (overall) | 1. Guangdong; 2. Beijing and its surrounding areas (such as Shanxi, Inner Mongolia, Hebei, and Tianjin) | Guangdong, Shanxi, and Inner Mongolia | Shanxi, Hebei, Tianjin, Inner Mongolia, and Beijing. | The fastest declining province after the intervention was Guangdong, while the fastest rising provinces after the intervention were Hebei and Tianjin; decreased by 16.7% compared with before intervention. |
| Internal flow (provincial flow) | 1. Guangdong; 2. Beijing and surrounding areas (such as Shanxi, Inner Mongolia, Hebei, and Tianjin) | Guangdong, Shanxi, and Inner Mongolia | Shanxi, Inner Mongolia, Hebei, Tianjin, and Beijing. | The fastest declining province after the intervention was Guangdong, while the fastest rising provinces after the intervention were Hebei, Tianjin and Beijing; decreased by 17.1% compared with before intervention. |
| Self-spreading flow (external flow) | Guangdong and Shanxi. | Guangdong, Shanxi, and Ningxia. | Guangdong, Shanxi, Hebei, Tianjin, Ningxia, and Shanghai. | After the intervention, Guangdong and Ningxia decreased rapidly; Shanxi, Hebei, Tianjin, Shanghai, and Ningxia rose faster; decreased by 2.6% compared with before intervention. |
| Hospitalized flow (external flow) | Hebei | Shanxi, Guangdong and Ningxia. | Hebei, Beijing, and Anhui. | After the intervention, Guangdong, Shanxi, Ningxia, and Shandong decreased rapidly; Hebei, Beijing, Anhui, Jilin, and Henan rose fastest, increased by 15.8% compared with before intervention. |
| Migrant flow (external flow) | Hebei, Sichuan, Guangxi, and Hunan. | Hebei, Sichuan, Guangxi, and Hunan. | Hebei, Beijing, Anhui, and Inner Mongolia. | After the intervention, Sichuan, Guangxi, and Hunan decreased more rapidly, Hebei, Beijing, Anhui, and Inner Mongolia, rose faster; decreased by 34.1% compared with before the intervention. |
