# Supplementary material for: Spatial pattern of severe acute respiratory syndrome in-out flow in 2003 in Mainland China
Source: BMC Infect Dis. 2014 Dec 31;14:721. doi: 10.1186/s12879-014-0721-y (PMC4322810; doi:10.1186/s12879-014-0721-y)
Supplement: Supplementary file 3 — Additional file 3: Table S3.: Sample size of processed data at the provincial level. (DOC 66 KB) [file 12879_2014_721_MOESM3_ESM.doc]

**Table S3 Sample size of processed data at the provincial level**

| **Province** | **Sample size** | **Current residence** | | **Onset location** | | **Report unit** | |
| --- | --- | --- | --- | --- | --- | --- | --- |
|  |  | **Valid data** | **Missing data** | **Valid data** | **Missing data** | **Valid data** | **Missing**  **data** |
| Beijing | 2489 | 1918 | 571 | 108 | 2381 | 1913 | 5 |
| Tianjin | 175 | 174 | 1 | 165 | 10 | 175 | 0 |
| Hebei | 226 | 223 | 3 | 220 | 6 | 226 | 0 |
| Shanxi | 452 | 446 | 6 | 420 | 32 | 420 | 32 |
| Inner Mongolia | 286 | 279 | 7 | 281 | 5 | 286 | 0 |
| Liaoning | 6 | 6 | 0 | 6 | 0 | 6 | 0 |
| Jilin | 35 | 34 | 1 | 35 | 0 | 35 | 0 |
| Heilongjiang | 0 | 0 | 0 | 0 | 0 | 0 | 0 |
| Shanghai | 8 | 8 | 0 | 8 | 0 | 8 | 0 |
| Jiangsu | 7 | 7 | 0 | 7 | 0 | 7 | 0 |
| Zhejiang | 4 | 4 | 0 | 4 | 0 | 4 | 0 |
| Anhui | 10 | 10 | 0 | 10 | 0 | 10 | 0 |
| Fujian | 3 | 3 | 0 | 3 | 0 | 3 | 0 |
| Jiangxi | 1 | 1 | 0 | 1 | 0 | 1 | 0 |
| Shandong | 1 | 1 | 0 | 1 | 0 | 1 | 0 |
| Henan | 15 | 15 | 0 | 15 | 0 | 15 | 0 |
| Hubei | 7 | 7 | 0 | 7 | 0 | 7 | 0 |
| Hunan | 6 | 5 | 1 | 6 | 0 | 6 | 0 |
| Guangdong | 1497 | 1460 | 37 | 609 | 888 | 1493 | 4 |
| Guangxi | 42 | 42 | 0 | 42 | 0 | 42 | 0 |
| Sichuan | 19 | 19 | 0 | 19 | 0 | 19 | 0 |
| Chongqing | 3 | 3 | 0 | 3 | 0 | 3 | 0 |
| San'xi | 12 | 10 | 2 | 12 | 0 | 12 | 0 |
| Gansu | 8 | 8 | 0 | 8 | 0 | 8 | 0 |
| Qinghai | 0 | 0 | 0 | 0 | 0 | 0 | 0 |
| Ningxia | 6 | 6 | 0 | 6 | 0 | 6 | 0 |
| Xinjiang | 0 | 0 | 0 | 0 | 0 | 0 | 0 |
| Sum | 5318 | 4689 | 629 | 1996 | 3322 | 4706 | 41 |
