# Supplementary material for: Spatial pattern of severe acute respiratory syndrome in-out flow in 2003 in Mainland China
Source: BMC Infect Dis. 2014 Dec 31;14:721. doi: 10.1186/s12879-014-0721-y (PMC4322810; doi:10.1186/s12879-014-0721-y)
Supplement: Supplementary file 4 — Additional file 4: Interpretation of typical types of SARS external flow. (Figure S1. SARS SSEs of SARS external flow. Figure S2. The first self-spreading flow of SARS. Figure S3. The second self-spreading flow of SARS. Figure S4. The first hospitalized flow of SARS. Figure S5. The second hospitalized flow of SARS. Figure S6. The first migrant flow of SARS. Figure S7. The second migrant flow of SARS.). (DOC 701 KB) [file 12879_2014_721_MOESM4_ESM.doc]

**Interpretation of typical types of SARS external flow**

**SSEs**

A SARS patient causing infection in >10 cases was called an SSE by the World Health Organization. According to available information, there were four SSEs that occurred across the regions [1-5], such as from Guangdong to Hong Kong, from Guangdong to Shanxi, from Shanxi to Beijing, from Beijing to Tianjin.


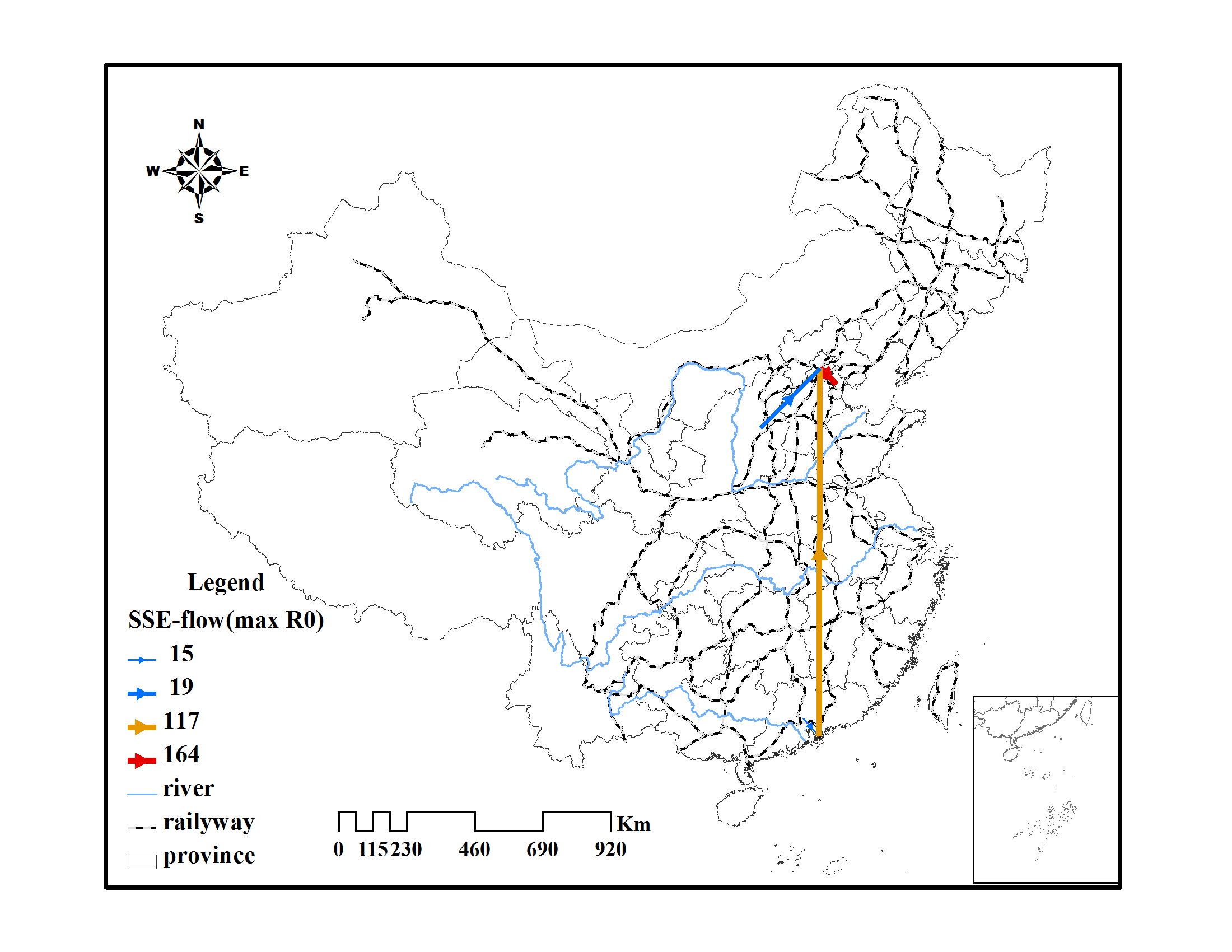


**Figure S1. SARS SSEs of SARS external flow**

**Self-spreading flow**


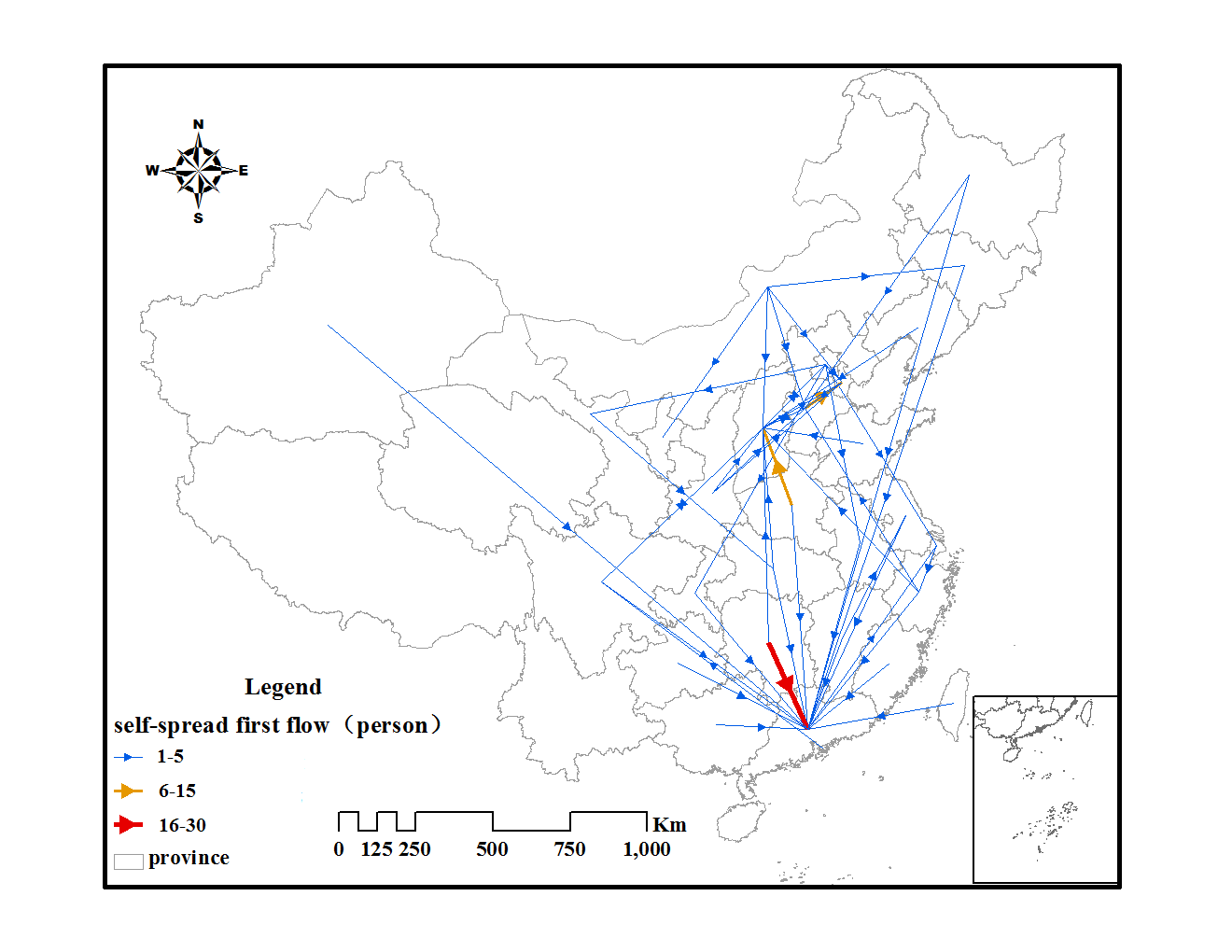


**Figure S2. The first self-spreading flow of SARS**

The first self-spreading flow of SARS, mainly from Hunan to Guangdong, accounting for 10.9% of SARS cases, followed by Hebei to Tianjin, Henan to Shanxi, and Henan to Guangdong, accounting for 6.9 %, 5.9% and 5.0% of cases, respectively. In addition, flow from Taiwan to Guangdong, and Hong Kong to Sichuan, accounted for 1.0% of cases each. The second self-spreading flow in the remaining provinces was more sporadic, especially in Tibet, Qinghai, Yunnan and Hainan, where there were no SARS cases of self-spreading flow.


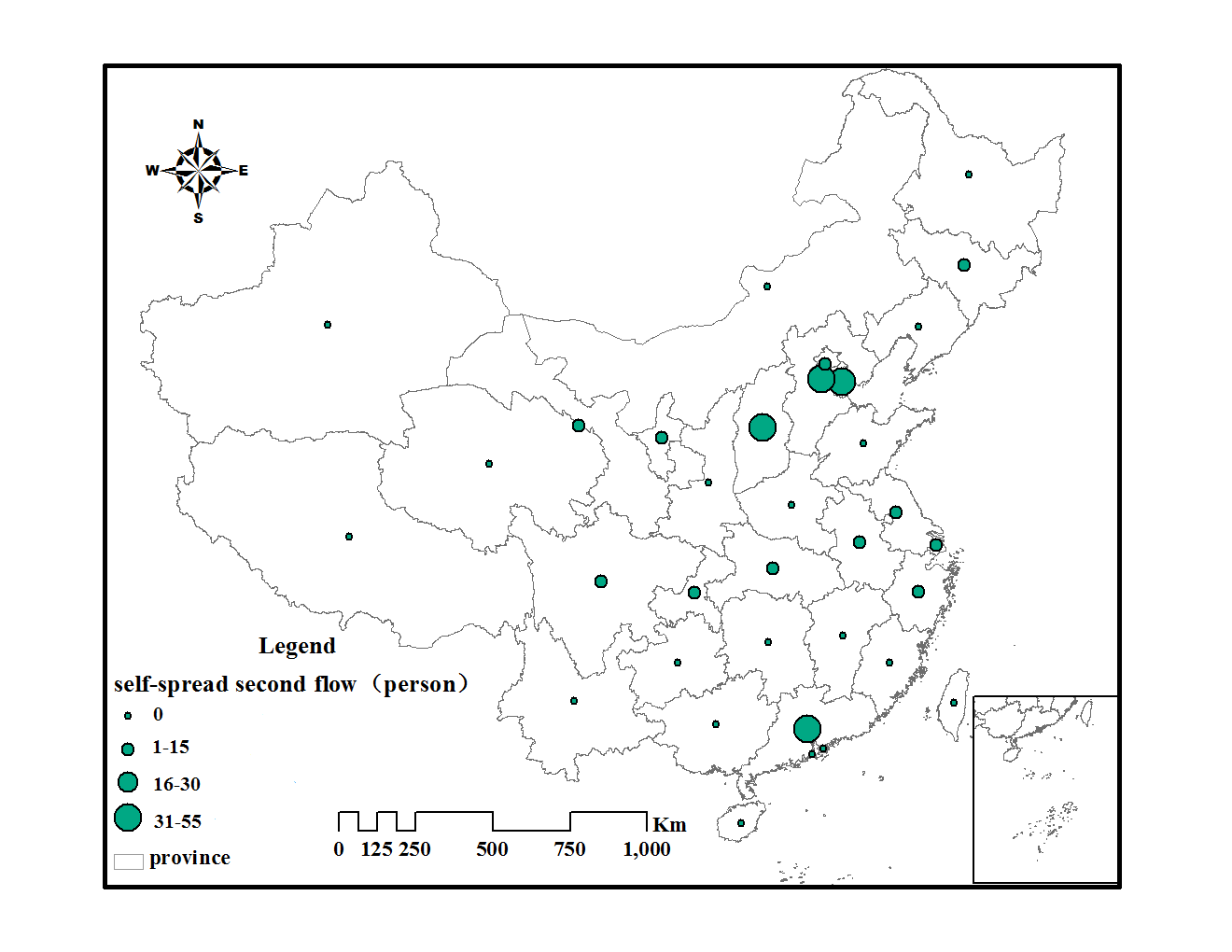


**Figure S3. The second self-spreading flow of SARS**

The second self-spreading flow of SARS was mainly within Guangdong and Shanxi, accounting for 36.6% and 20.8% of cases, respectively, followed by Tianjin, Shanghai and Hebei, accounting for 10.9%, 9.9% and 5.0% of cases, respectively. In addition, a small amount of self-spreading flow accounted for 16.8% of cases in 10 provinces: Ningxia, Gansu, Anhui, Sichuan, Beijing, Hubei, Jilin, Jiangsu, Zhejiang and Sichuan.

**Hospitalized flow**


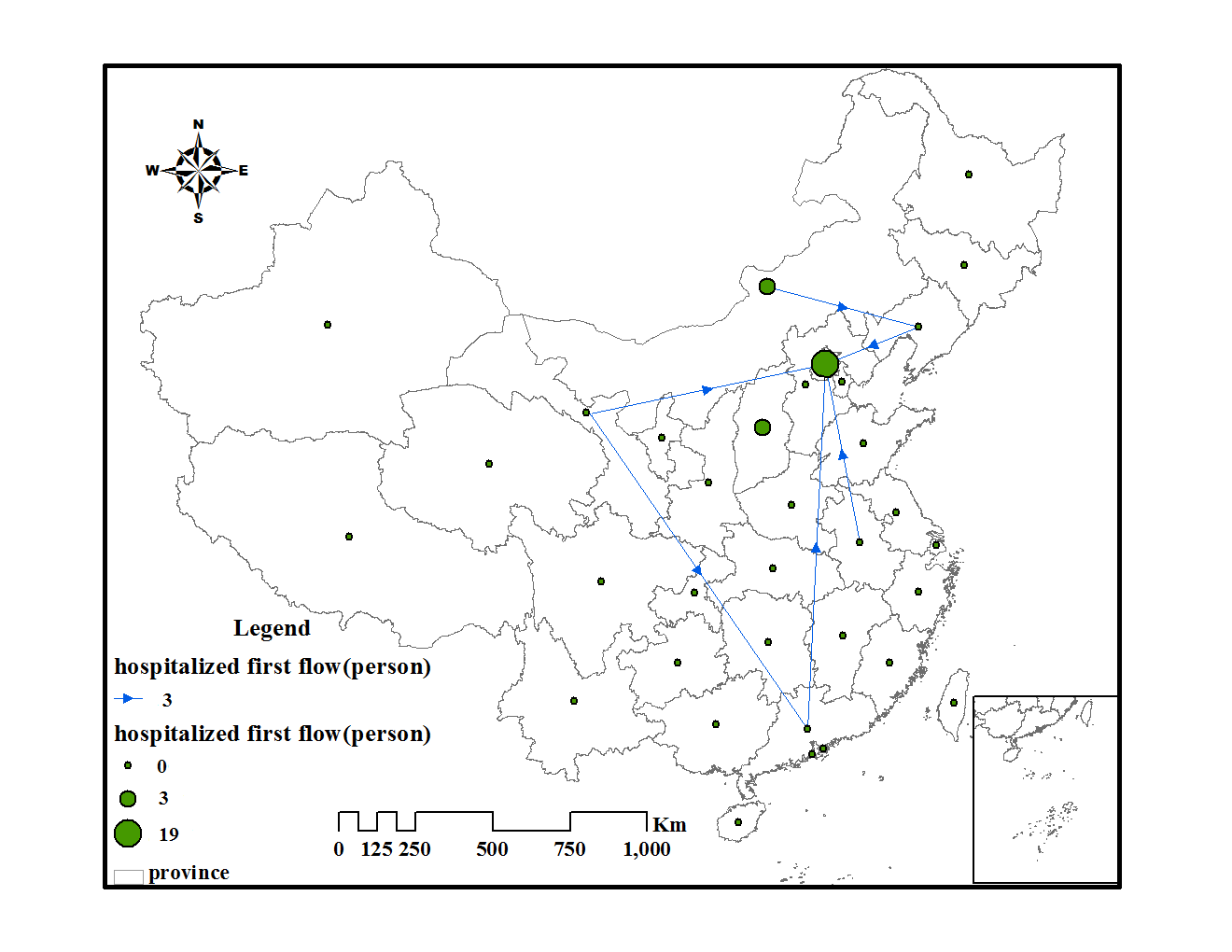


**Figure S4. The first hospitalized flow of SARS**

The first hospitalized flow of SARS was mainly in Beijing, accounting for 46.7% of cases, followed by Inner Mongolia and Shanxi, and from Anhui to Beijing, Gansu to Beijing, Guangdong to Beijing, Liaoning to Beijing, Gansu to Guangdong, and Inner Mongolia to Liaoning, accounting for 6.7% of cases, respectively. In addition, there were no cases in other provinces.


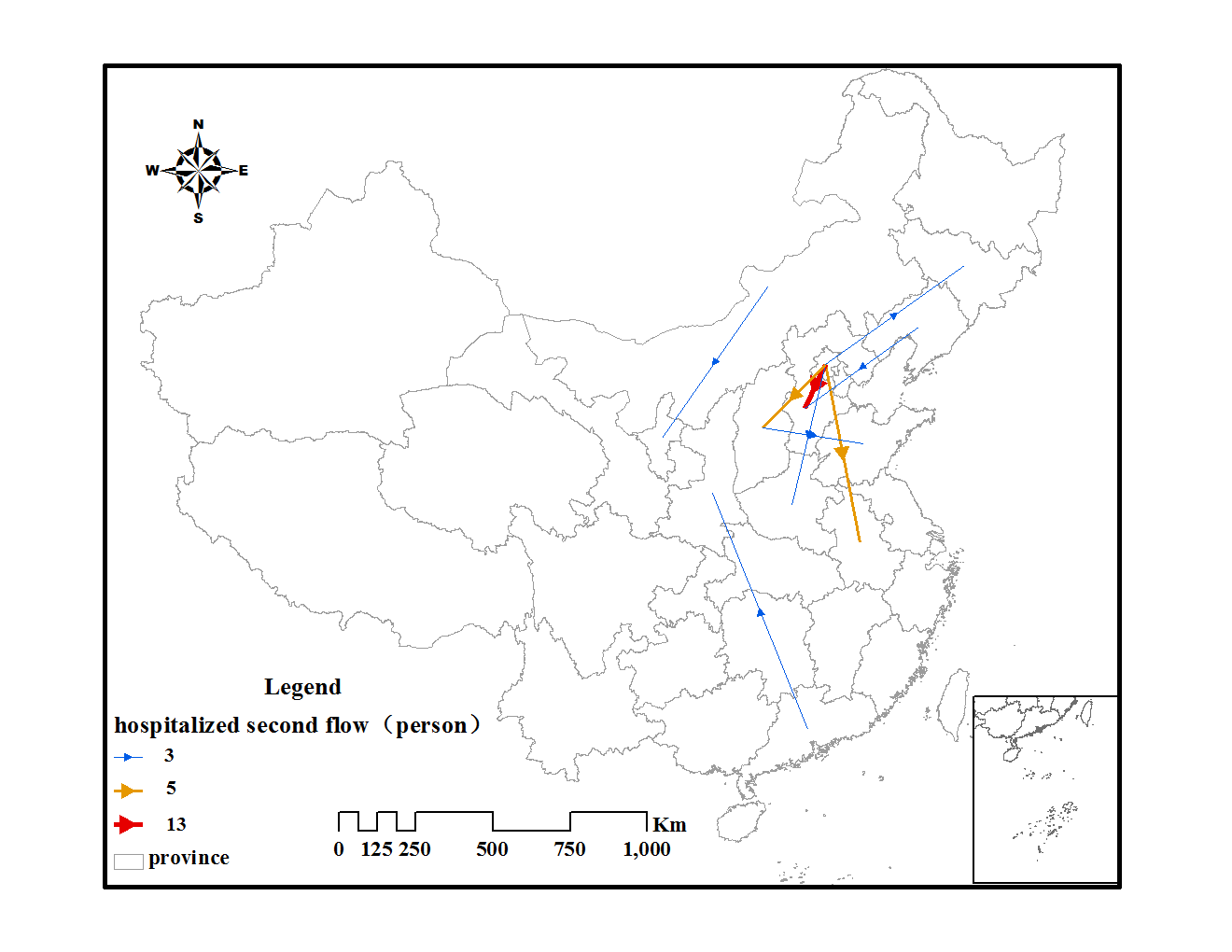


**Figure S5. The second hospitalized flow of SARS**

The second hospitalized flow of SARS was mainly from Beijing to Hebei, accounting for 33.3% of cases, followed by Beijing to Anhui and Beijing to Shanxi, accounting for a total of 26.6% of cases. Other flows occurred from Beijing to Henan, Beijing to Jilin, Guangdong to Shaanxi, Liaoning to Hebei, Inner Mongolia to Ningxia, and Shanxi to Shandong, accounting for 6.7% of cases, respectively.

**Migrant flow**


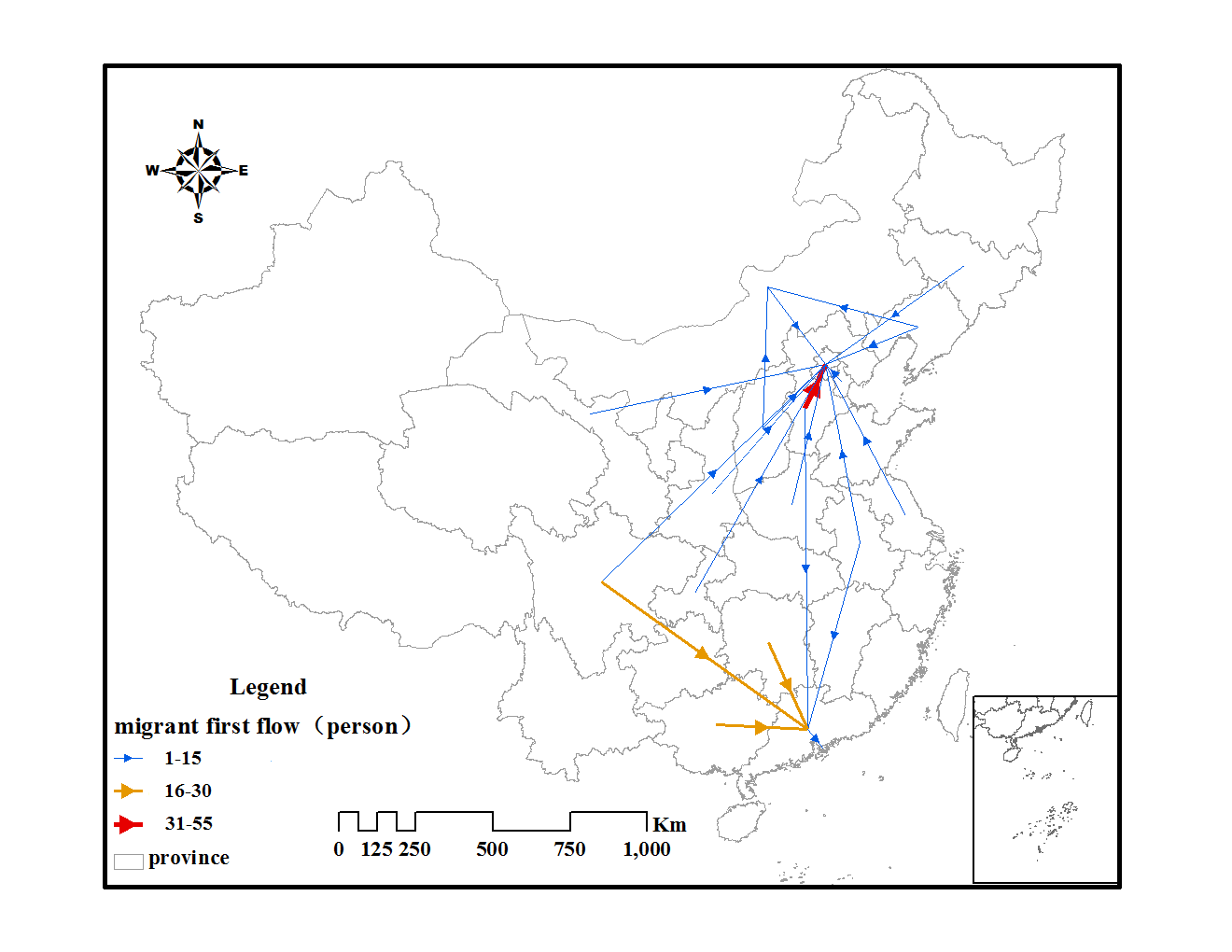


**Figure S6. The first migrant flow of SARS**

The first migrant flow of SARS was mainly from Hebei to Beijing, accounting for 30.5% of cases, followed by Guangxi to Guangdong, Sichuan to Guangdong, and Hunan to Guangdong, accounting for 11.0%, 8.5% and 7.3% of cases, respectively. In addition, the first migrant flow between Hong Kong and Guangdong accounted for 4.9% of cases. The number of cases was more dispersed in other regions, especially in Xinjiang, Tibet, Qinghai, Yunnan, Guizhou, Hubei, Hainan, Jiangxi, Fujian, Taiwan, Zhejiang, Shandong, Ningxia and Heilongjiang, where no first migrant flow were found.


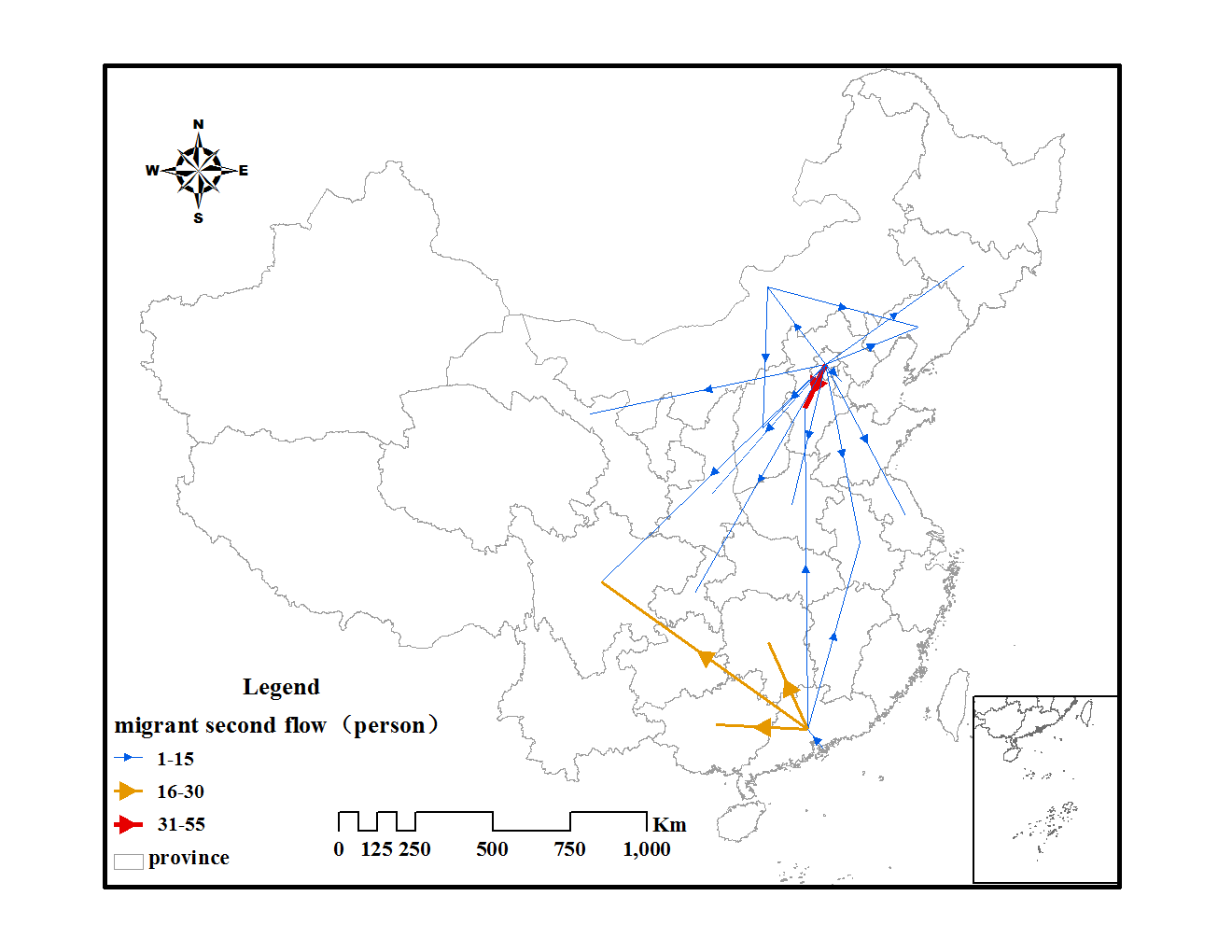


**Figure S7. The second migrant flow of SARS**

The second migrant flow of SARS was mainly from Beijing to Hebei, accounting for 30.5% of cases, followed by Guangdong to Guangxi, Guangdong to Sichuan, and Guangdong to Hunan, accounting for 11.0%, 8.5%, and 7.3% of cases, respectively. In addition, the second migrant flow between Hong Kong and Guangdong accounted for 4.9% of cases. The number of cases was more dispersed in other regions, especially in Xinjiang, Tibet, Qinghai, Yunnan, Guizhou, Hubei, Hainan, Jiangxi, Fujian, Taiwan, Zhejiang, Shandong, Ningxia and Heilongjiang, where there were no second migrant flow were found.

**References**

1. Li Q, Zeng G, Ou JM, Guo GP: **Investigation and analysis on one case of SARS transmission chain**. *Chinese Medical Journal* 2003, **83**(11):906-909.

2. Liu LY, Zhou JF: **Transmission chain analysis on the first SARS case in Huairou District of Beijing**. *Chinese Journal of Public Health* 2003, **19**(9):1032-1032.

3. Liu ZJ, Shen Z, He X, Huang RG, Teng RM, Ning F, Li XM, Ding LX, Lin CY: **Epidemiology analysis on a input case of SARS in Beijing**. *Chinese Journal of Epidemiology* 2003, **24**(5):358-359.

4. Wang XX, Hong HY, Liu D, Zhang ZL, Dan AL, Zhu XJ, Gao ZG, Wang XD, Xia YY, Chen X: **SARS epidemic characteristics and main control measures evaluation in Tianjin**. *Chinese Journal of Epidemiology* 2003, **24**(7):565-569.

5. Wang YQ, Wang XH: **Research and analysis on the first SARS epidemic transmission chain in Dongcheng District of Beijing**. *Disease Surveillance* 2004, **19**(9):349-350.
