# Supplementary figures and images for: Spatial pattern of severe acute respiratory syndrome in-out flow in 2003 in Mainland China
Source: BMC Infect Dis. 2014 Dec 31;14:721. doi: 10.1186/s12879-014-0721-y (PMC4322810; doi:10.1186/s12879-014-0721-y)

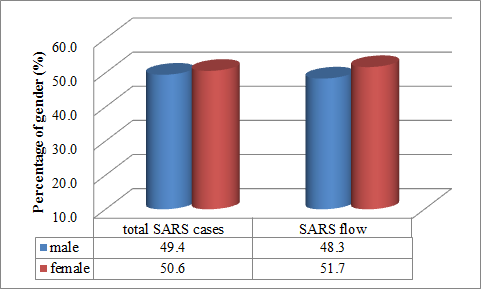


**Figure S8. Frequency distributions of the SARS flow and total SARS cases by gender**

Supplement: Supplementary file 5 — Additional file 5: Figure S8.: Frequency distributions of the SARS flow and total SARS cases by gender. (DOC 50 KB) [file 12879_2014_721_MOESM5_ESM.doc]

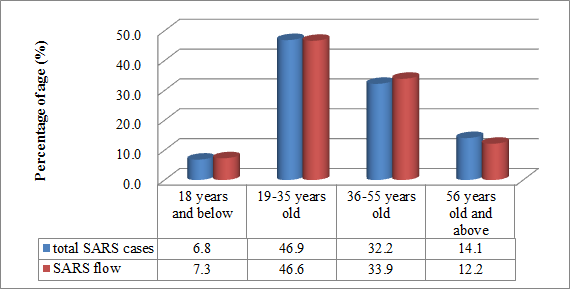


**Figure S9. Frequency distributions of the SARS flow and total SARS cases by age**

Supplement: Supplementary file 6 — Additional file 6: Figure S9.: Frequency distributions of the SARS flow and total SARS cases by age. (DOC 52 KB) [file 12879_2014_721_MOESM6_ESM.doc]

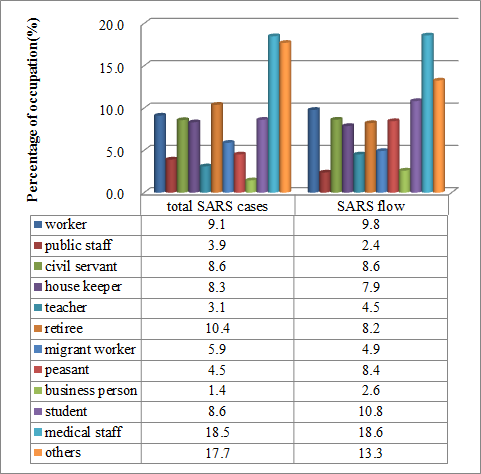


**Figure S10. Frequency distributions of the SARS flow and total SARS cases by occupations**

Supplement: Supplementary file 7 — Additional file 7: Figure S10.: Frequency distributions of the SARS flow and total SARS cases by occupations. (DOC 59 KB) [file 12879_2014_721_MOESM7_ESM.doc]

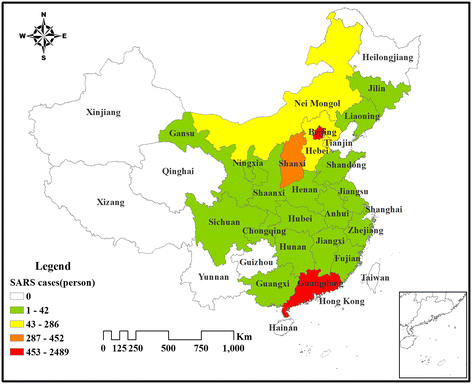

Supplement: Supplementary file 9 — Authors’ original file for figure 1 [file 12879_2014_721_MOESM9_ESM.gif]

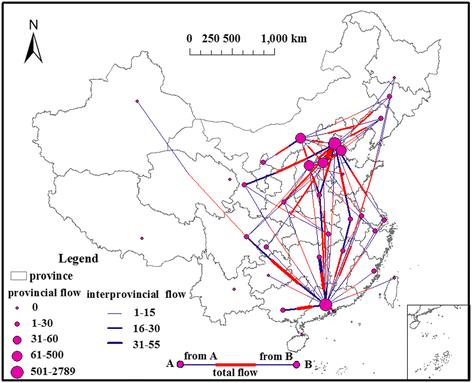

Supplement: Supplementary file 10 — Authors’ original file for figure 2 [file 12879_2014_721_MOESM10_ESM.gif]

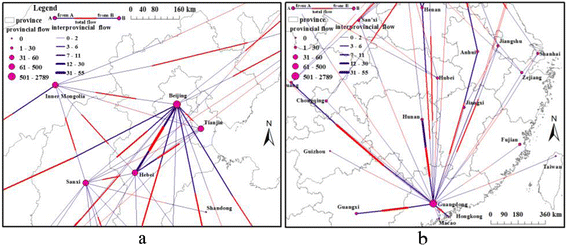

Supplement: Supplementary file 11 — Authors’ original file for figure 3 [file 12879_2014_721_MOESM11_ESM.gif]

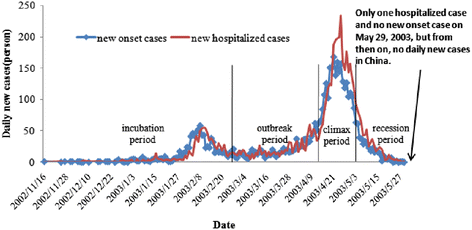

Supplement: Supplementary file 12 — Authors’ original file for figure 4 [file 12879_2014_721_MOESM12_ESM.gif]

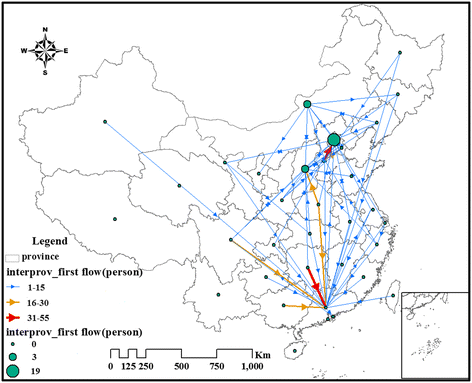

Supplement: Supplementary file 13 — Authors’ original file for figure 5 [file 12879_2014_721_MOESM13_ESM.gif]

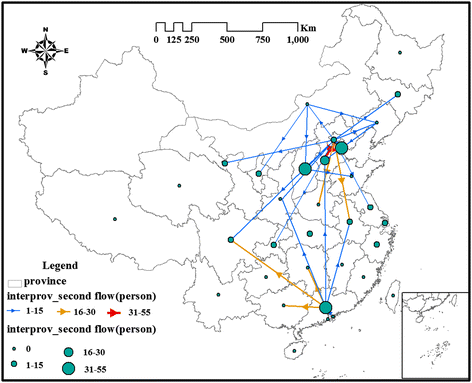

Supplement: Supplementary file 14 — Authors’ original file for figure 6 [file 12879_2014_721_MOESM14_ESM.gif]

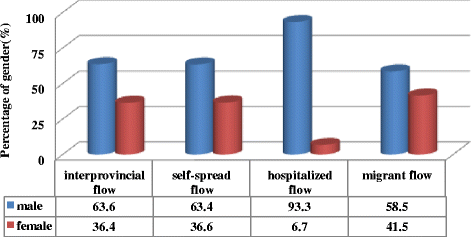

Supplement: Supplementary file 15 — Authors’ original file for figure 7 [file 12879_2014_721_MOESM15_ESM.gif]

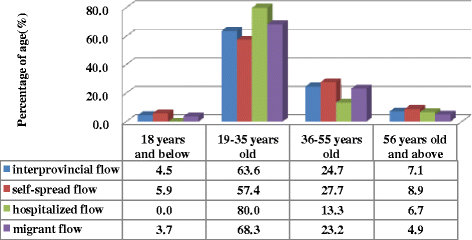

Supplement: Supplementary file 16 — Authors’ original file for figure 8 [file 12879_2014_721_MOESM16_ESM.gif]

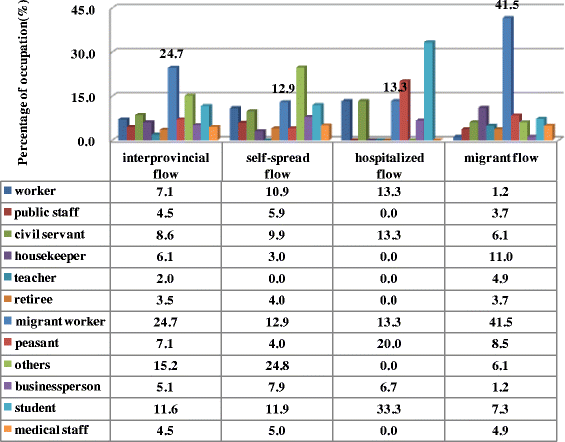

Supplement: Supplementary file 17 — Authors’ original file for figure 9 [file 12879_2014_721_MOESM17_ESM.gif]
