# Supplementary material for: Spatial pattern of severe acute respiratory syndrome in-out flow in 2003 in Mainland China
Source: BMC Infect Dis. 2014 Dec 31;14:721. doi: 10.1186/s12879-014-0721-y (PMC4322810; doi:10.1186/s12879-014-0721-y)
Supplement: Supplementary file 8 — Additional file 8: Ratio estimator is sampling with proportional to aggregate size, which is used to update the total in-out flow. (DOC 34 KB) [file 12879_2014_721_MOESM8_ESM.doc]

**Ratio estimator**

Ratio estimator is sampling with proportional to aggregate size [1], which is used to update the total in-out flow *y*, it is expressed by the formula:

$$y=\frac{x}{1-\rho}$$

where *x* denotes the number of the cases of provincial flow or interprovincial flow in a province; *ρ* denotes the percentage of missing data of provincial flow or interprovincial flow in the province. When there are no SARS cases in one province, *ρ* = 1*.*

**References**

1. Cochran WG: **Sampling Techniques**. *Wiley* 1977 ( third edition).
